# Supplementary material for: Selective hydrodeoxygenation of α, β-unsaturated carbonyl compounds to alkenes
Source: Nat Commun. 2024 Mar 9;15:2166. doi: 10.1038/s41467-024-46383-9 (PMC10925037; doi:10.1038/s41467-024-46383-9)
Supplement: Supplementary file 1 — Supplementary Information [file 41467_2024_46383_MOESM1_ESM.pdf]

Supplementary Information

Selective hydrodeoxygenation of α, β-unsaturated carbonyl compounds to alkenes

Tianjiao Wang[1, 2], Yu Xin[1, 2], Bingfeng Chen[1], Bin Zhang[1], Sen Luan[1, 2], Minghua Dong[1, 2], Yuxuan Wu[1, 2], Xiaomeng Cheng[1, 2], Ye Liu[1], Huizhen Liu[1, 2*], Buxing Han[1, 2]

[1]Beijing National Laboratory for Molecular Sciences, CAS Key Laboratory of Colloid and Interface and Thermodynamics, CAS Research/Education Center for Excellence in Molecular Sciences, Center for Carbon Neutral Chemistry, Institute of Chemistry, Chinese Academy of Sciences, Beijing 100190, China.
[2]School of Chemistry and Chemical Engineering, University of Chinese Academy of Sciences, Beijing 100049, China.
[*]email: liuhz@iccas.ac.cn

Supplementary Tables

Supplementary Table 1. Elemental analysis of ZnNC-X catalysts.
Supplementary Table 2. Effect of the hydrogen source.
Supplementary Table 3. Brönsted acidic site density, Lewis acidic site density, total acidity content and the ratio of B/L of ZnNC-X catalysts.
Supplementary Table 4. Effect of additives on reaction.
Supplementary Table 5. The content of the various N species in ZnNC-X catalysts.
Supplementary Table 6. Effect of the hydrogen source and KSCN.
Supplementary Table 7. The fitting curve parameters for Zn K-edge EXAFS for ZnNC-900 catalyst.

Supplementary Figures

Supplementary Figure 1. Schematic illustration of the preparation process of ZnNC-X catalysts.
Supplementary Figure 2. SEM images of ZnNC-X catalysts.
Supplementary Figure 3. SEM image of ZnNC-900 catalyst.
Supplementary Figure 4. TEM image and EDS elemental mapping images of ZnNC-900 catalyst.
Supplementary Figure 5. XRD patterns of ZnNC-900 catalyst, Zn and ZnO.
Supplementary Figure 6. Effect of reaction temperature of Zn/NC-900 catalyst.
Supplementary Figure 7. Effect of reaction time of ZnNC-X catalysts.
Supplementary Figure 8. Optimization of reaction conditions for the hydrodeoxygenation over Zn/NC-900 catalyst.
Supplementary Figure 9. FTIR spectra of ZnNC-900 catalyst and NC-900 catalyst.
Supplementary Figure 10. Mass spectrum of the by-product by GC-MS.
Supplementary Figure 11. Mass spectra of the by-products by LC-MS.
Supplementary Figure 12. Characterization of acid-base properties on ZnNC-X catalysts.
Supplementary Figure 13. Pyridine-FTIR spectra of ZnNC-X catalysts.
Supplementary Figure 14. ^{1}H NMR spectra of the reaction solution.
Supplementary Figure 15. FTIR spectra of CAL adsorption on ZnNC-600 catalyst.
Supplementary Figure 16. Chromatogram of the gas collected after reaction.
Supplementary Figure 17. N 1*s* XPS spectra for Zn/NC-X catalysts.
Supplementary Figure 18. Optimization of reaction conditions over Zn/NC-900 catalyst.
Supplementary Figure 19. Kinetic plots of the hydrodeoxygenation of triphenylmethanol to triphenylmethane.
Supplementary Figure 20. Mass fragmentation analysis of triphenylmethane.
Supplementary Figure 21. Zn K-edge EXAFS (points) and the fitting curve (line) for ZnNC-900 catalyst, shown in k^3-weighted k-space.
Supplementary Figure 22. Zn K-edge EXAFS (points) and the fitting curves (line) for ZnNC-900 catalyst, shown in R-space.
Supplementary Figure 23. The possible reaction mechanism.

Supplementary data

Supplementary Tables

Supplementary Table 1. Elemental analysis of ZnNC-X catalysts.

Catalysts	C (wt%)	N (wt%)	H (wt%)	Zn (wt%)
ZnNC-500	41.8	24.4	4.3	19.8
ZnNC-600	36.4	22.2	2.2	18.4
ZnNC-700	36.8	19.8	1.8	17.0
ZnNC-800	43.7	16.1	2.5	14.5
ZnNC-900	54.0	11.0	2.2	9.5
ZnNC-1000	76.1	6.4	1.1	5.4

The content of zinc element in catalysts was measured by inductively coupled plasma optical emission spectrometer (ICP-OES). The content of C, H and N elements in catalysts was measured by organic element analyzer.

Supplementary Table 2. Effect of the hydrogen source.

Entry	Reaction conditions		Conv. (%)	Y_{1c} (%)
	Sol.	Gas		
1	TBA	H_2	30.4	-
2	IPA	N_2	95.0	78.2

Reaction conditions: cinnamaldehyde (0.2 mmol), ZnNC-900 (20 mg), solvent (4 ml), 1 MPa, 150 °C, 11 h. The conversion of substrate and the yield of products were determined by GC with dodecane as internal standard.

Supplementary Table 3. Brönsted acidic site density, Lewis acidic site density, total acidity content and the ratio of B/L of ZnNC-X catalysts.

Catalysts	T (°C)	BAS[a] density (μmol/g)	LAS[b] density (μmol/g)	Total acidity (μmol/g)	B/L[c]
ZnNC-500	100	10.5	153.4	163.9	0.068
	200	8.7	87.0	95.7	0.099
ZnNC-600	100	11.1	187.1	198.2	0.059
	200	8.7	112.4	121.1	0.077
ZnNC-700	100	4.0	107.5	111.5	0.037
	200	1.8	50.0	46.7	0.039
ZnNC-800	100	7.5	97.3	104.	0.077
	200	5.2	79.3	84.5	0.066
ZnNC-900	100	13.8	166.6	180.4	0.083
	200	6.9	111.2	118.1	0.062
ZnNC-1000	100	8.1	114.4	122.5	0.070
	200	6.8	77.3	84.2	0.088

[a] Brönsted acidic site (BAS). [b] Lewis acidic site (LAS). [c] Molar ratio of BAS-to-LAS. The BAS density and LAS density were calculated based on the corresponding peak area of pyridine-FTIR.

Supplementary Table 4. Effect of additives on reaction.

Entry	Substrates	Additives	Conv. (%)	Sel. (%)			
				1c	1d	1e+1f	1g
1[a]	CAL	-	95.0	88.2	-	-	-
2[a]	CAL	boric acid	70.3	52.5	-	-	-
3[a]	CAL	pyridine	72.0	44.2	-	-	-
4[a]	CAL	KSCN	53.7	36.8	-	-	-
5[b]	COL	-	99.9	-	11.0	75.7	4.6
6[b]	COL	boric acid	84.8	-	11.9	81.6	-
7[b]	COL	pyridine	78.5	-	13.9	84.7	-
8[b]	COL	KSCN	22.2	-	27.2	45.4	-
9[c]	CAL	-	99.9	5.6	10.9	61.1	2.5
10[c]	CAL	boric acid	99.9	18.5	10.1	55.8	-
11[c]	CAL	pyridine	99.9	7.0	8.9	60.9	-
12[c]	CAL	KSCN	99.9	54.0	9.5	14.1	-

Reaction conditions: substrates (0.2 mmol), ZnNC-900 (20 mg), IPA (4 mL), N_2 (1 MPa), additives (20 mg). [a] 150°C, 11 h; [b] 180°C, 7 h; [c] 180 °C, 24 h. The conversion of substrate and the yield of products were determined by GC with dodecane as internal standard.

Supplementary Table 5. The content of the various N species in ZnNC-X catalysts.

Catalysts	Pyridinic-N (%)	Zn-N_x (%)	Pyrrolic-N (%)	Graphitic-N (%)	Oxidized-N (%)
ZnNC-500	96.6	0.3	6.6	0.0	0.5
ZnNC-600	83.5	0.2	12.3	3.7	0.3
ZnNC-700	74.3	10.6	7.4	4.9	2.8
ZnNC-800	60.7	17.2	11.3	6.3	4.5
ZnNC-900	49.9	18.7	11.9	10.7	8.9
ZnNC-1000	42.5	10.1	19.0	19.1	9.4

The content of various N species in catalyst was obtained by the peak area of XPS.

Supplementary Table 6. Effect of the hydrogen source and KSCN.

Entry	Reaction conditions			Conv. (%)	Y. triphenylmethane (%)
	Gas	Additives	Sol.		
1	N_2	-	IPA	45.9	43.0
2	H_2	-	IPA	44.1	43.8
3	H_2	-	TBA	8.2	6.8
4	N_2	KSCN	IPA	5.0	3.8

Reaction conditions: triphenylmethanol (30 mg), ZnNC-900 (20 mg), gas (1 MPa), solvent (2 mL), 140 °C, 4 h, KSCN (20 mg). The conversion of substrates and the yield of products were determined by GC with dodecane as internal standard.

Supplementary Table 7. The fitting curve parameters [a] for Zn K-edge EXAFS for ZnNC-900 catalyst.

Path	d^b/Å	N	R/Å	σ^2/Å^2
Zn-N	1.932	3.9(6)	2.01(1)	0.004(1)

[a] S_0^2 was fixed as 0.93, ΔE_0 was refined as a global fit parameter, returning a value of (2±1) eV. Data ranges: $3.3 \leq k \leq 11.2$ Å^{-1}, $1.2 \leq R \leq 2.35$ Å. The number of variable parameters is 4, out of a total of 5.5 independent data points. R facter for this fit is 0.7%. [b] The distance for Zn-N is from the FEEF file.

Supplementary Figures

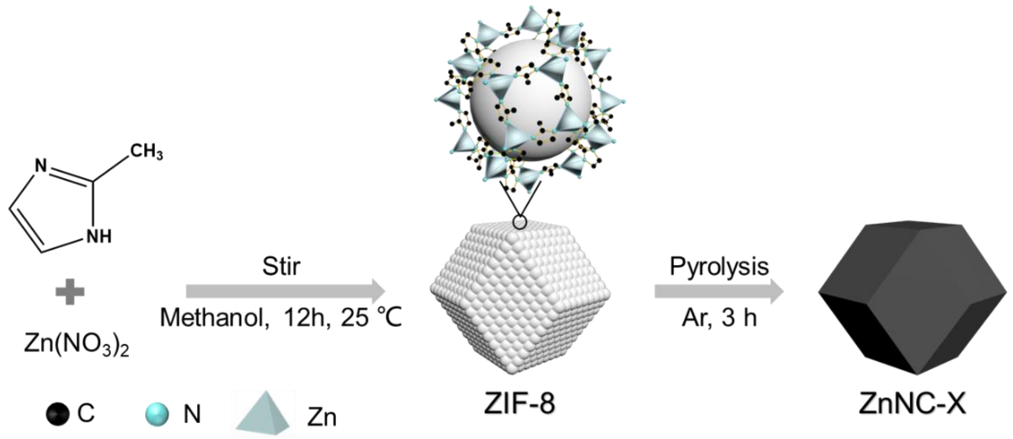

Supplementary Figure 1. Schematic illustration of the preparation process of ZnNC-X catalysts.

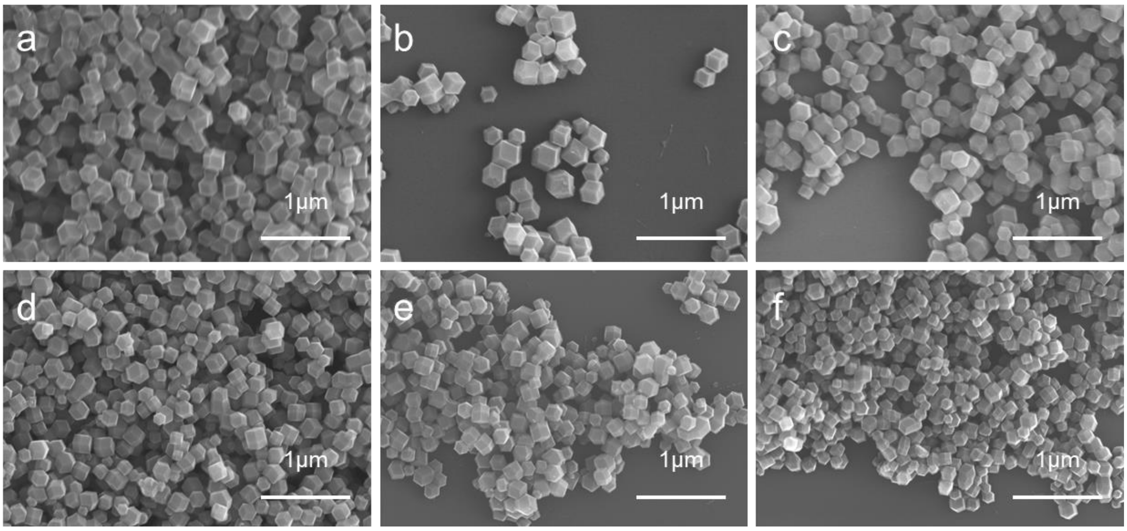

Supplementary Figure 2. SEM images of ZnNC-X catalysts. a ZIF-8. **b** ZnNC-500. **c** ZnNC-600. **d** ZnNC-700. **e** ZnNC-800. **f** ZnNC-1000.

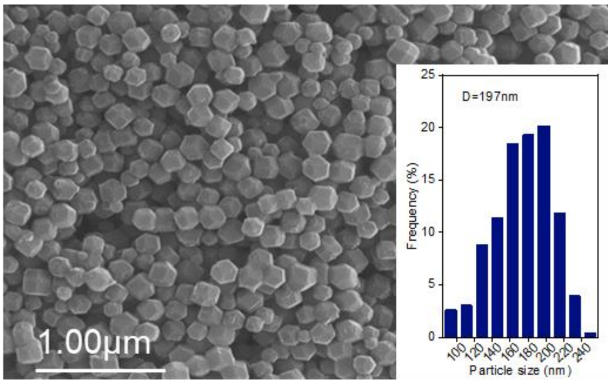

Supplementary Figure 3. SEM image of ZnNC-900 catalyst. The inset shows the particle size distributions of ZnNC-900 catalyst.

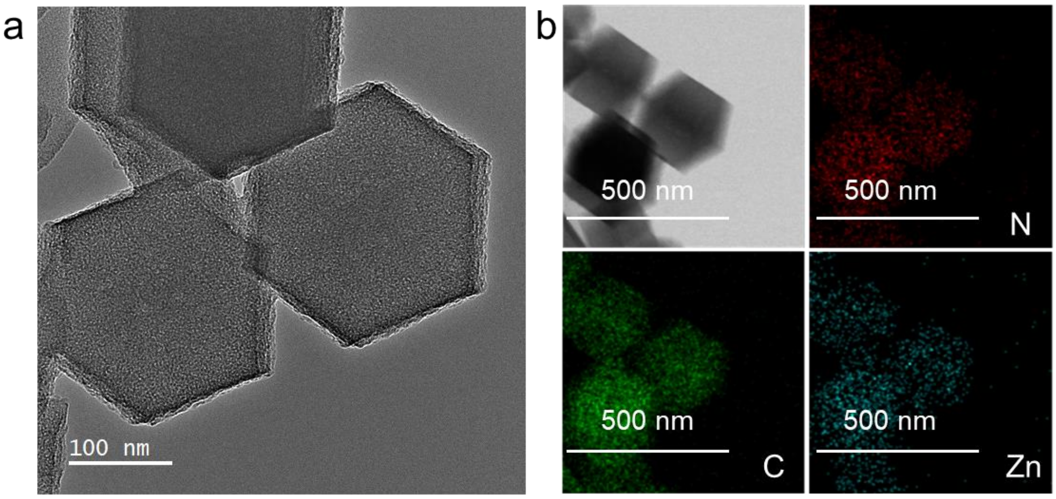

Supplementary Figure 4. TEM image and EDS elemental mapping images of ZnNC-900 catalyst. a TEM image. **b** N, C and Zn EDS elemental mapping images of ZnNC-900 catalyst (Red is N. Green is C. Blue is Zn.).

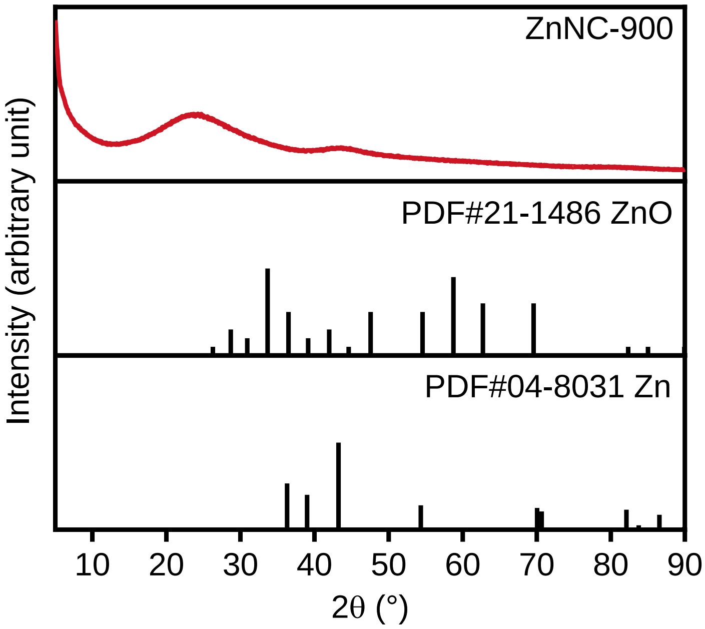

Supplementary Figure 5. XRD patterns of ZnNC-900 catalyst, Zn and ZnO.

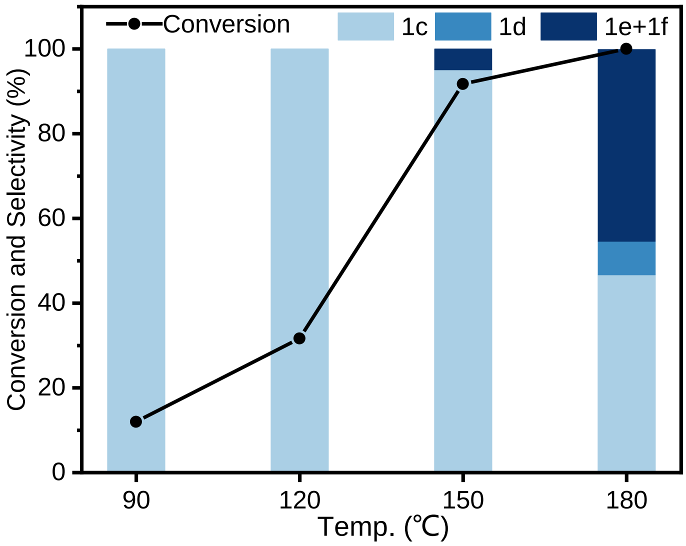

Supplementary Figure 6. Effect of reaction temperature of Zn/NC-900 catalyst. Reaction conditions: cinnamaldehyde (0.2 mmol), ZnNC-900 (20 mg), 2-propanol (4 mL), 11 h, N_2 (1 MPa).

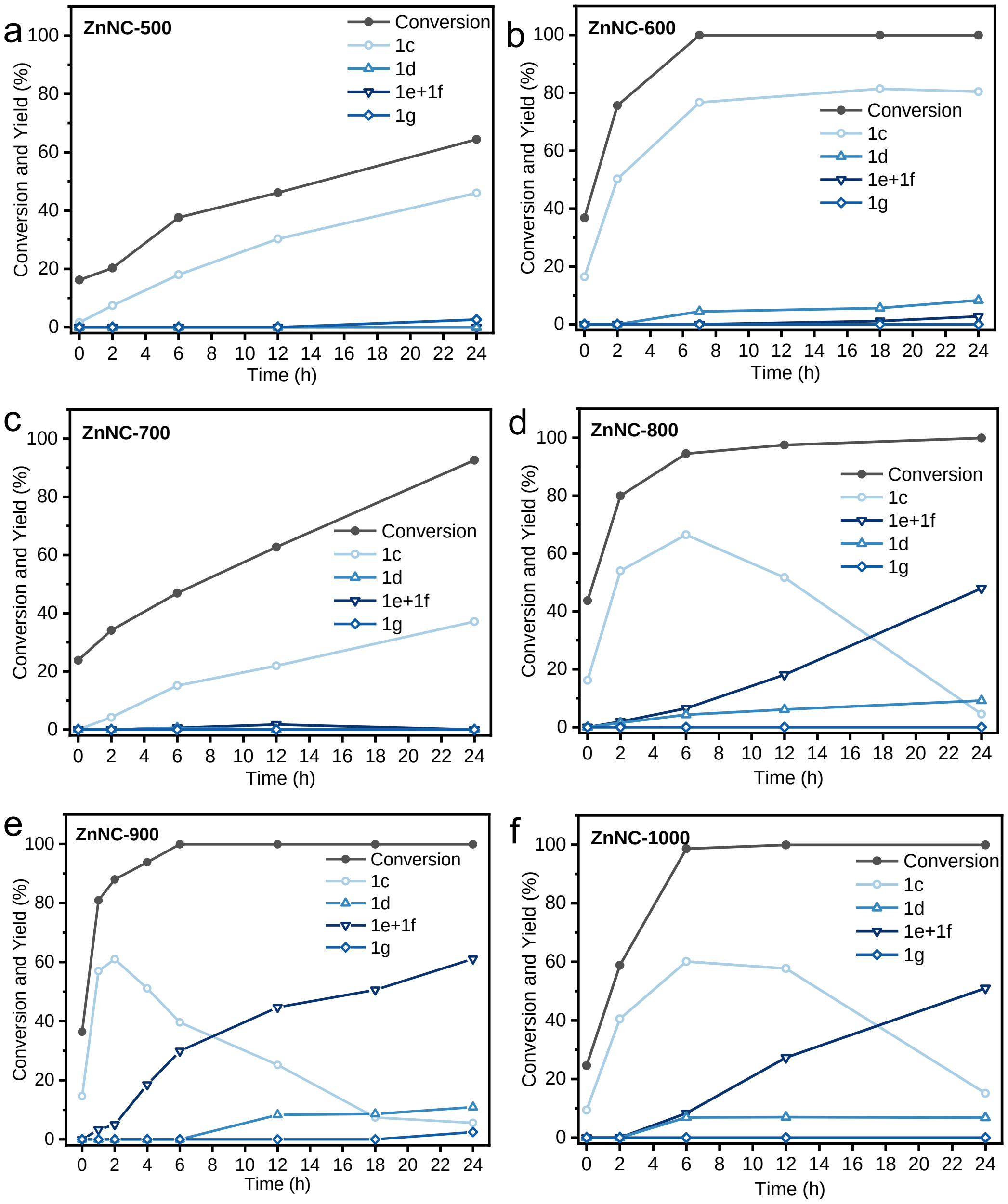

Supplementary Figure 7. Effect of reaction time of ZnNC-X catalysts. a ZnNC-500. **b** ZnNC-600. **c** ZnNC-700. **d** ZnNC-800. **e** ZnNC-900. **f** ZnNC-1000. **Reaction conditions**: cinnamaldehyde (0.2 mmol), ZnNC-X (20 mg), 2-propanol (4 mL), 180 °C, N_2 (1 MPa).

Supplementary Figure 7 displays the time curves for ZnNC-X (X=500-1000). It is evident from the data that the primary product catalyzed by ZnNC-X (X=500-600) was COL. In the case of ZnNC-X (X=800-1000), as the reaction time extended, the yield of COL initially rose and then declined. Concurrently, the production of alkenes continued to increase, and the rate of alkene formation accelerated after nearly complete conversion of the CAL substrate. It could be seen from the time curves that the presence of CAL partially inhibited the hydrodeoxygenation of COL (Supplementary Fig. 7c-f). This indicated that CAL and COL competed for adsorption at the same site (Zn-N_x site).

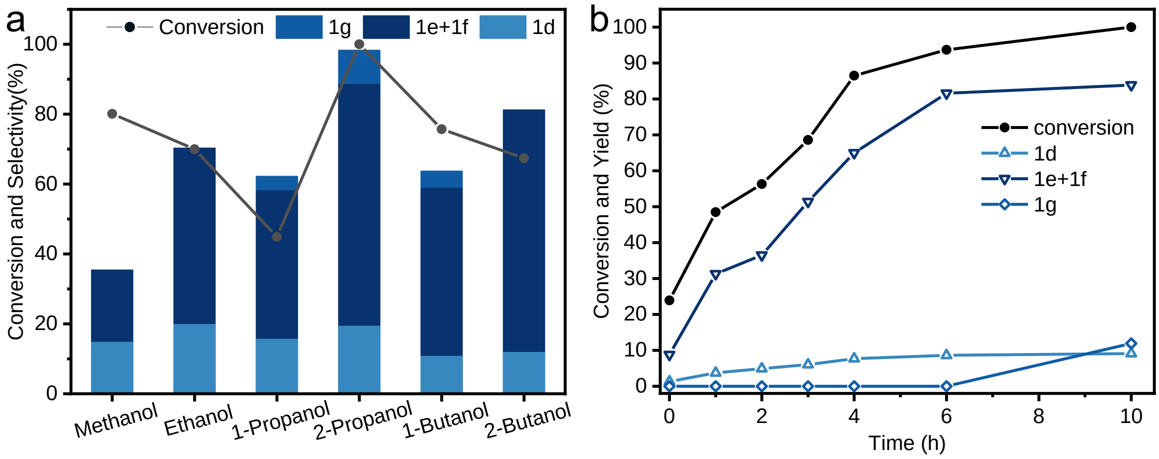

Supplementary Figure 8. Optimization of reaction conditions for the hydrodeoxygenation over Zn/NC-900 catalyst. a Effect of reaction solvent. **Reaction conditions**: cinnamyl alcohol (0.2 mmol), ZnNC-900 (20 mg), solvent (4 mL), N_2 (1 MPa), 180 °C, 7 h. **b** Effect of reaction time. **Reaction conditions**: cinnamyl alcohol (0.2 mmol), ZnNC-900 (20 mg), 2-propanol (4 mL), N_2 (1 MPa), 180 °C. The conversion of substrate and the yield of products were determined by GC with dodecane as internal standard.

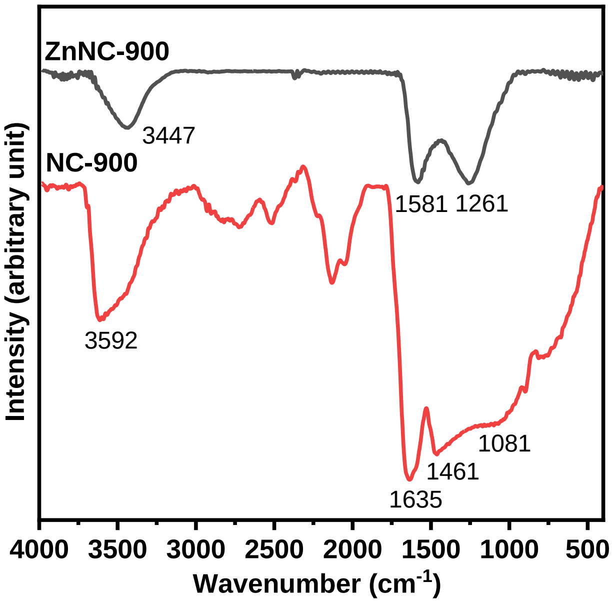

Supplementary Figure 9. FTIR spectra of ZnNC-900 catalyst and NC-900 catalyst.
The peaks observed at 3447 cm^{-1} and 3592 cm^{-1} correspond to the stretching vibrations of the OH group. The peaks located at 1635 cm^{-1} and 1580 cm^{-1} are attributed to the stretching vibrations of the C=X (X=C, O or N) bond.

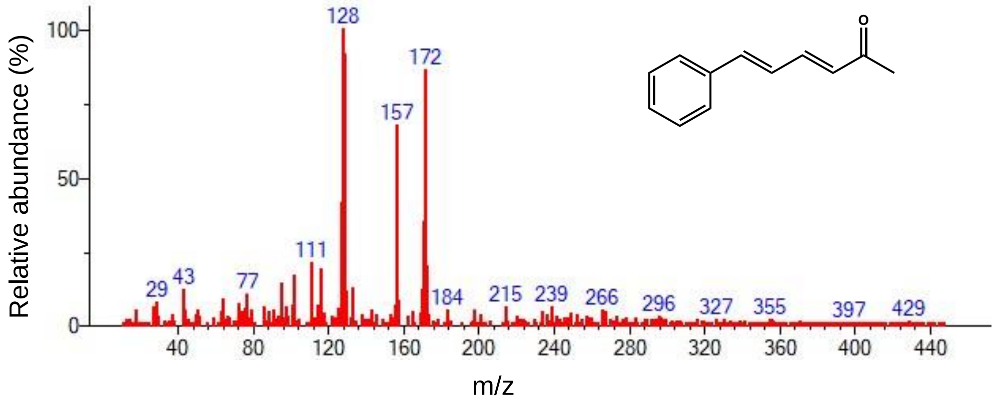

Supplementary Figure 10. Mass spectrum of the by-product by GC-MS (EI).
(E, E)-6-phenyl-3,5-hexadien-2-one, one of the by-products, was obtained by aldol condensation of cinnamaldehyde with acetone and then dehydration.

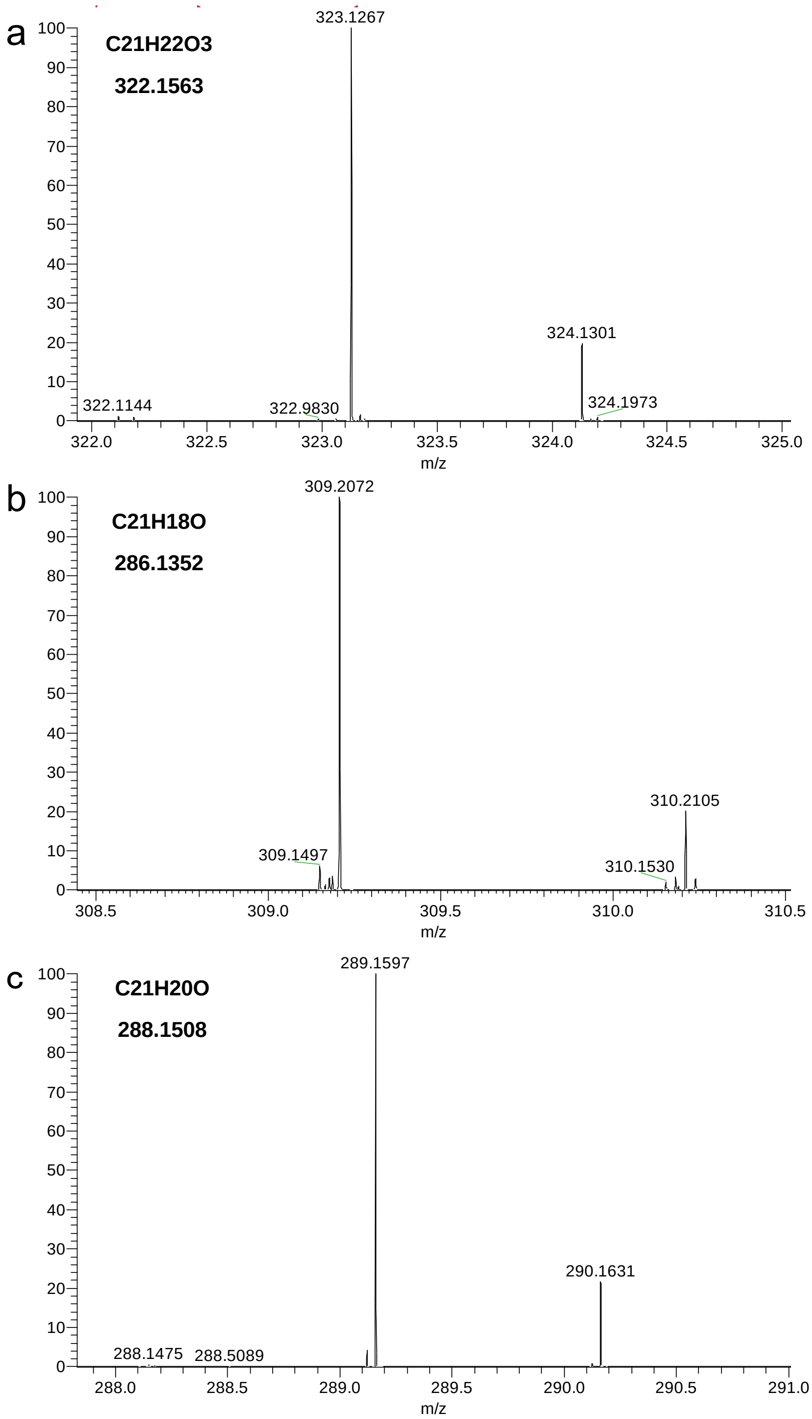

Supplementary Figure 11. Mass spectra of the by-products by LC-MS (ESI).

a $C_{21}H_{22}O_3$ LC-MS (ESI): m/z calcd for $C_{21}H_{23}O_3$ $[M+H]^+$:323.1641; found: 323.1267.

b $C_{21}H_{18}O$ LC-MS (ESI): m/z calcd for $C_{21}H_{18}ONa$ $[M+Na]^+$:309.1249; found: 309.2072.

c $C_{21}H_{20}O$ LC-MS (ESI): m/z calcd for $C_{21}H_{21}O$ $[M+H]^+$: 289.1587; found: 289.1597.

The by-products may be formed by aldol condensation of one molecule of acetone and two molecules of cinnamaldehyde.

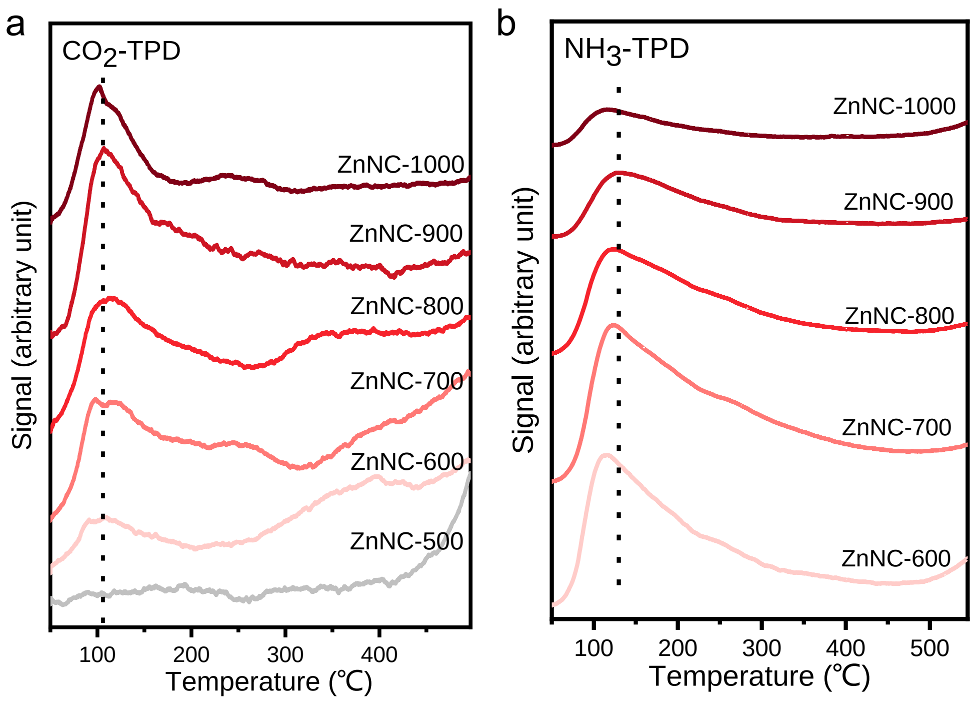

Supplementary Figure 12. Characterization of acid-base properties on ZnNC-X catalysts. a CO_2 temperature-programmed desorption (CO_2-TPD) profiles of ZnNC-X catalysts. **b** NH_3 temperature-programmed desorption (NH_3-TPD) profiles of ZnNC-X catalysts. Baseline drift after temperature above 400 °C may be due to the decomposition of catalysts.

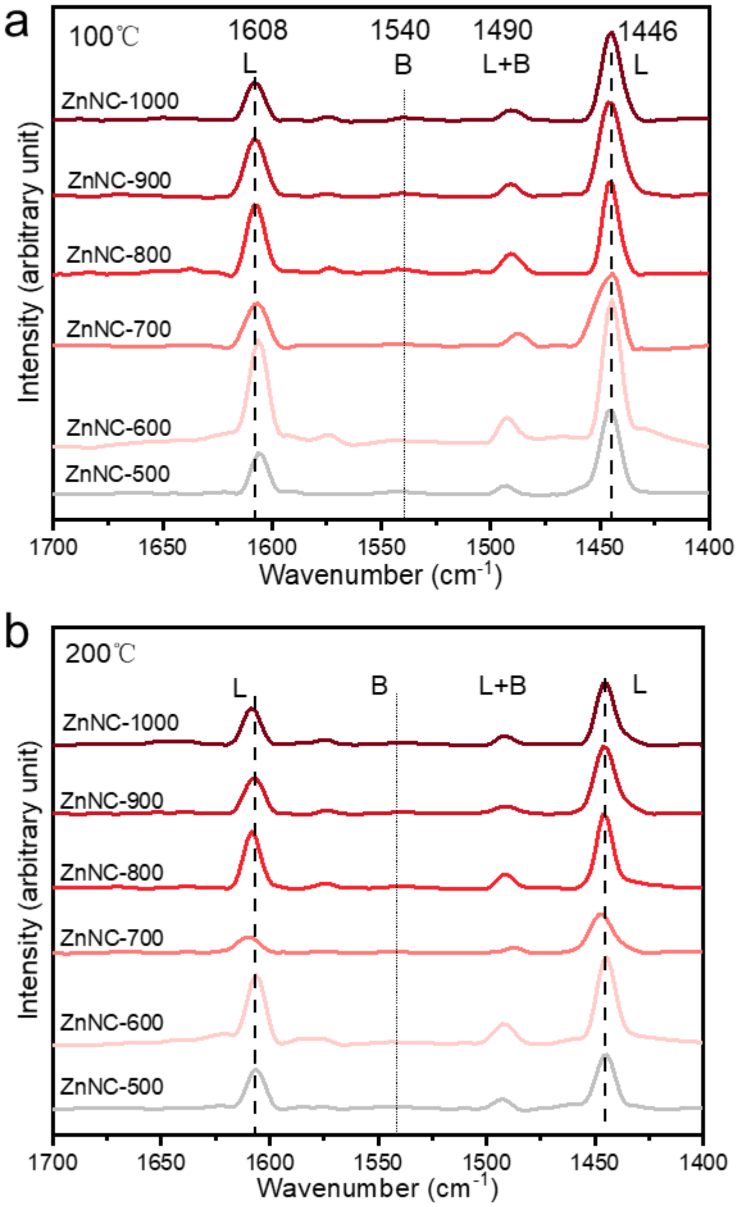

Supplementary Figure 13. Pyridine-FTIR spectra of ZnNC-X catalysts. a FTIR spectra of pyridine adsorption on ZnNC-X at 100 °C. **b** FTIR spectra of pyridine adsorption on ZnNC-X at 200 °C. (B: Brönsted acid; L: Lewis acid)

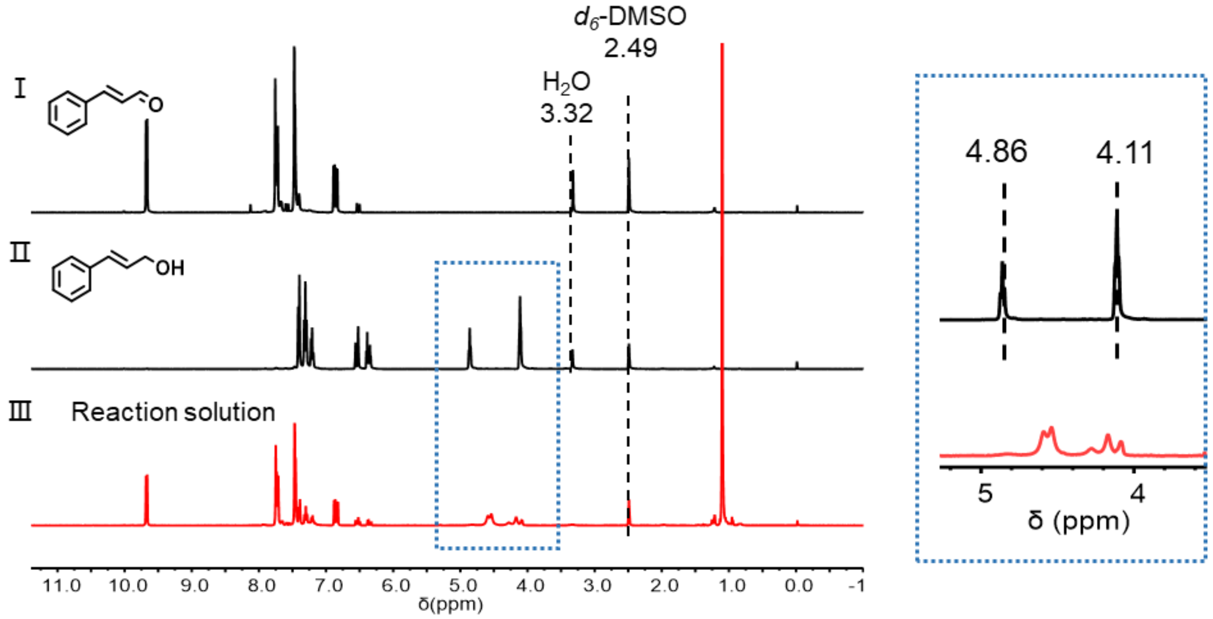

Supplementary Figure 14. ^{1}H NMR spectra of the reaction solution. **I** Cinnamaldehyde in d_6-DMSO. **II** Cinnamyl alcohol in d_6-DMSO. **III** Reaction solution in d_6-DMSO. **Reaction conditions**: cinnamaldehyde (0.2 mmol), ZnNC-900 (20 mg), 2-propanol-d_8 (1 mL), *t*-butanol, 150 °C, 11h, N_2 (1 MPa). The molar ratio of 2-propanol-d_8 to *t*-butanol is 1:3. Enlargement of the range from 3.5 to 5.2 ppm for the ^{1}H NMR spectra of the II.

4.86 ppm is the nuclear magnetic peak of the H on the hydroxyl group. 4.11 ppm is the nuclear magnetic peak of the two H on *α*-C. In Figure II, the ratio of the peak area at 4.86 ppm to that at 4.11 ppm is about 1:2. And correspondingly, in the reaction solution, the area ratio between these two peaks is about 1:1 since H on *α*- C changes to D.

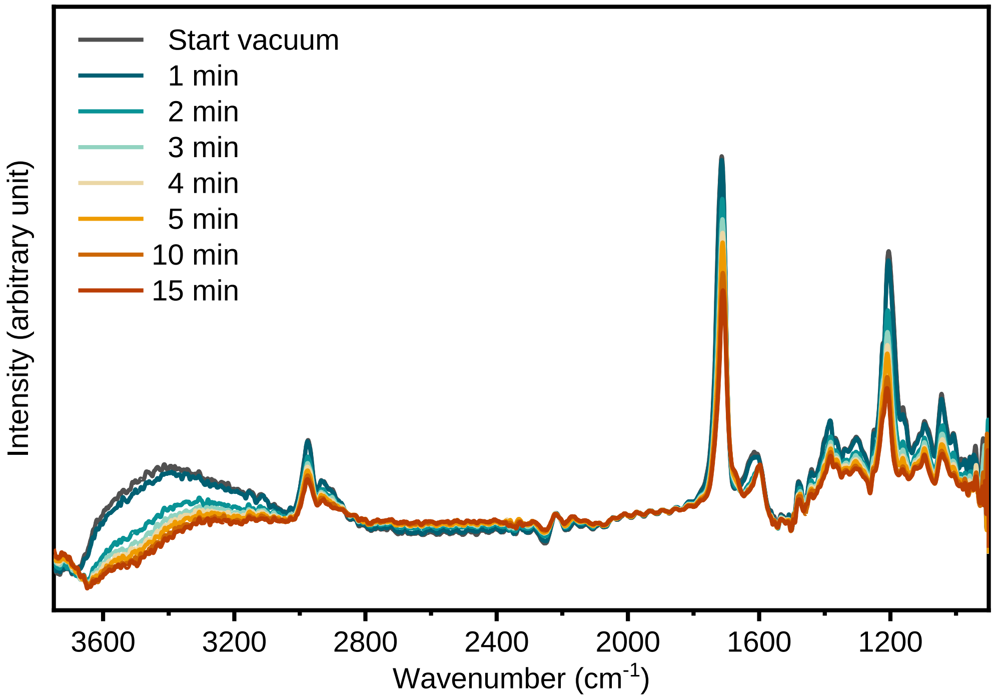

Supplementary Figure 15. FTIR spectra of CAL adsorption on ZnNC-600 catalyst. Figure 4a is a partial infrared spectrum of Supplementary Figure 15. 1610 cm^{-1} and 3600-3000 cm^{-1} are attributed to the infrared peaks of water.

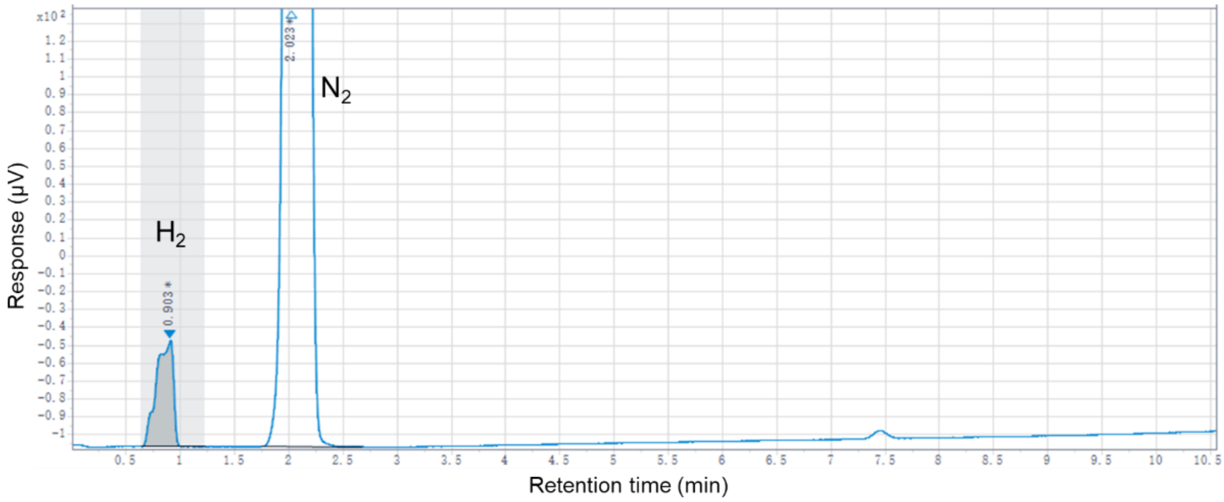

Supplementary Figure 16. Chromatogram of the gas collected after reaction.

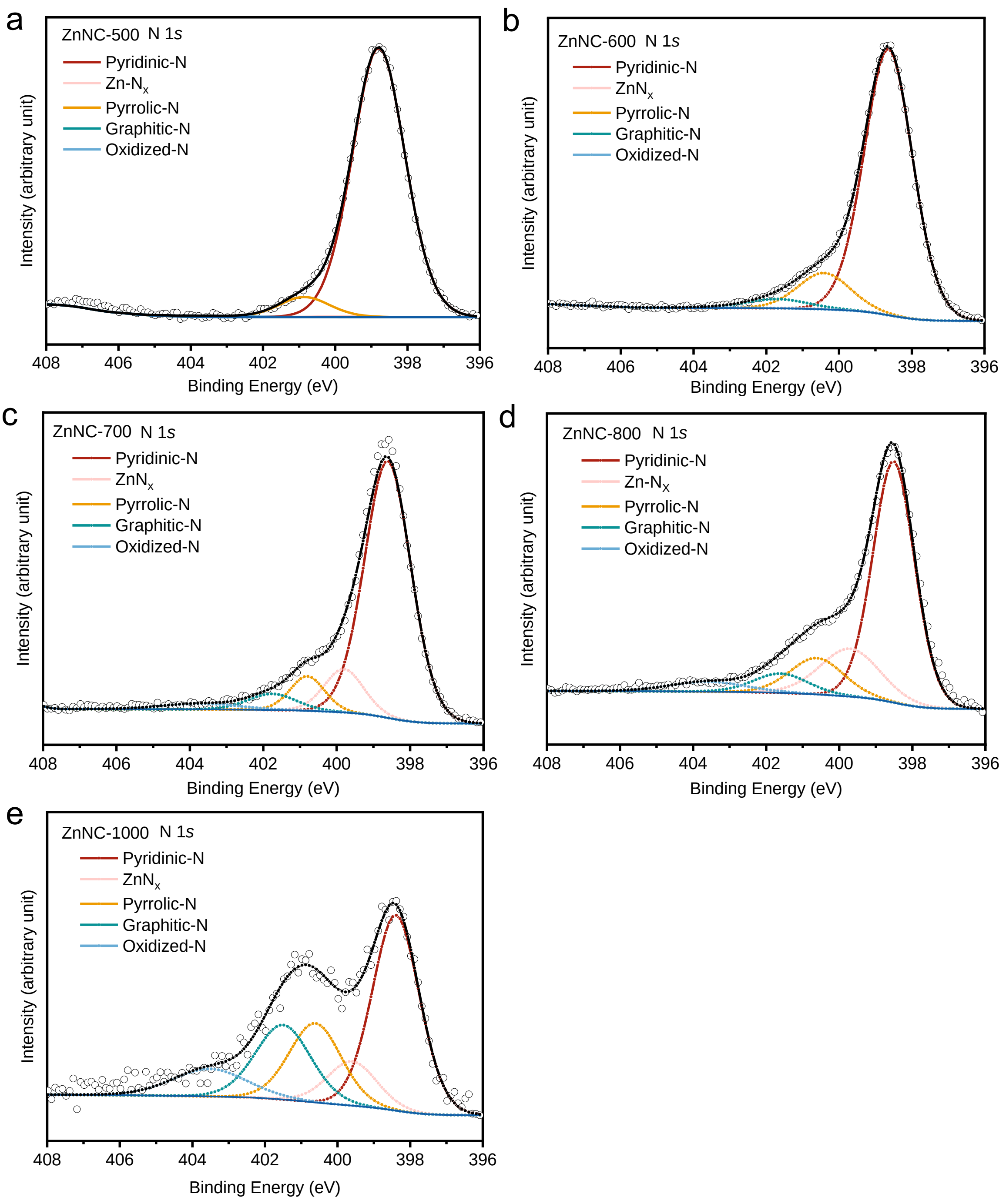

Supplementary Figure 17. N 1*s* XPS spectra for Zn/NC-X catalysts. a ZnNC-500. **b** ZnNC-600. **c** ZnNC-700. **d** ZnNC-800. **e** ZnNC-1000.

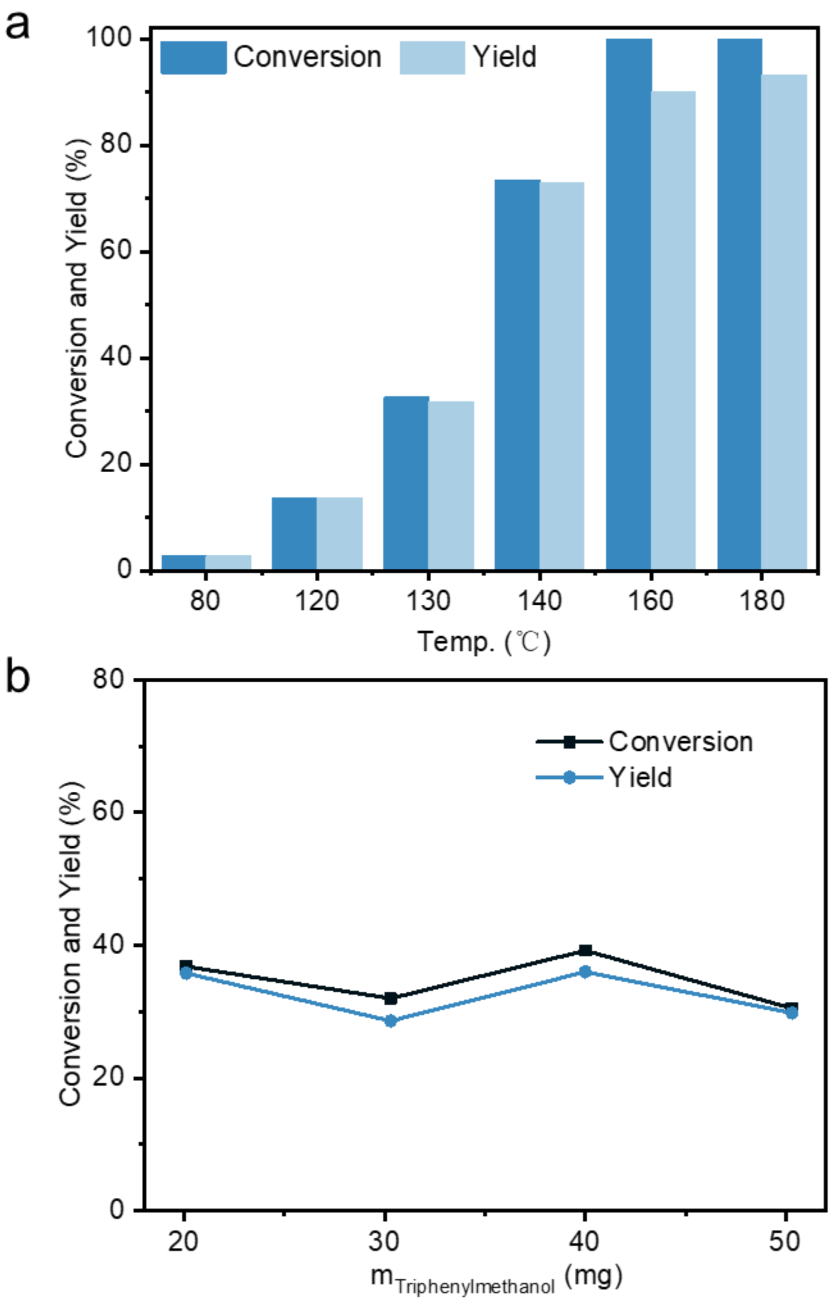

Supplementary Figure 18. Optimization of reaction conditions over Zn/NC-900 catalyst. a Effect of reaction temperature. **Reaction conditions**: triphenylmethanol (30 mg), ZnNC-900 (20 mg), 2-propanol (4 mL), N_2 (1 MPa), 7 h. **b** Effect of the amount of triphenylmethanol. **Reaction conditions**: ZnNC-900 (20 mg), 2-propanol (4 mL), N_2 (1 MPa), 140 °C, 3 h. The conversion of substrate and the yield of products were determined by GC with dodecane as internal standard.

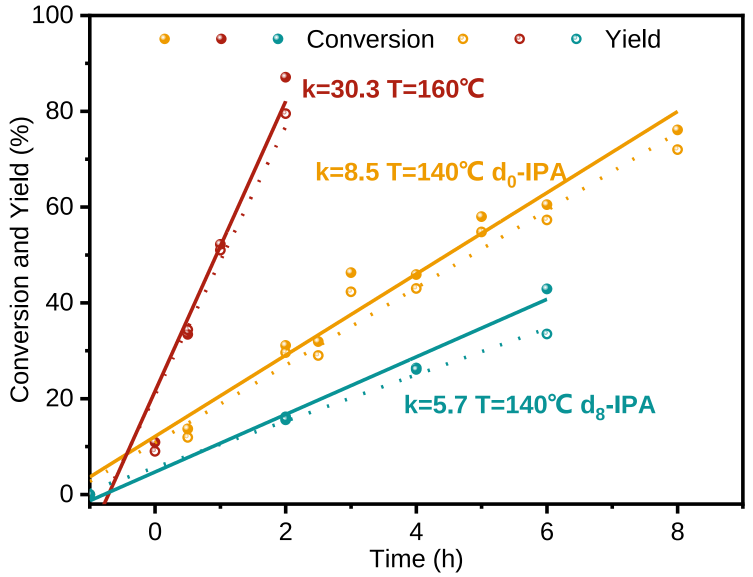

Supplementary Figure 19. Kinetic plots of the hydrodeoxygenation of triphenylmethanol to triphenylmethane. The lines and dashed lines are linear fits for conversion and yield, respectively. **Reaction conditions**: triphenylmethanol (30 mg), ZnNC-900 (20 mg), solvent (2 mL), N_2 (1 MPa). The conversion of substrate and the yield of products were determined by GC with dodecane as internal standard.

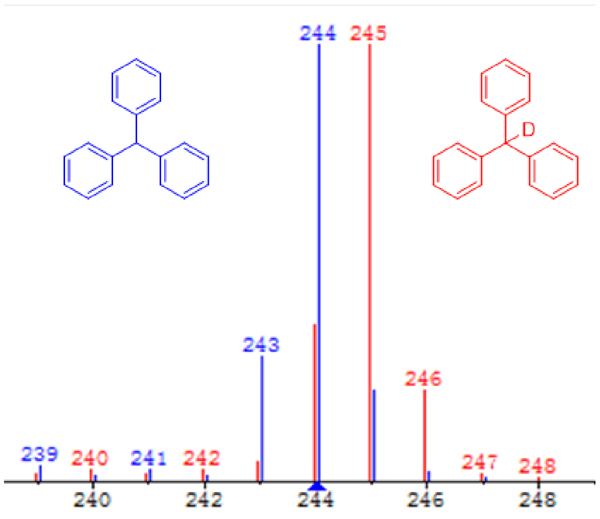

Supplementary Figure 20. Mass fragmentation analysis of triphenylmethane. Blue is the standard mass spectrum of triphenylmethane. Red is the mass spectrum of the reaction product. **Reaction conditions**: triphenylmethanol (30 mg), ZnNC-900 (20 mg), 2-propanol-d_8 (2 mL), N_2 (1 MPa), 8 h.

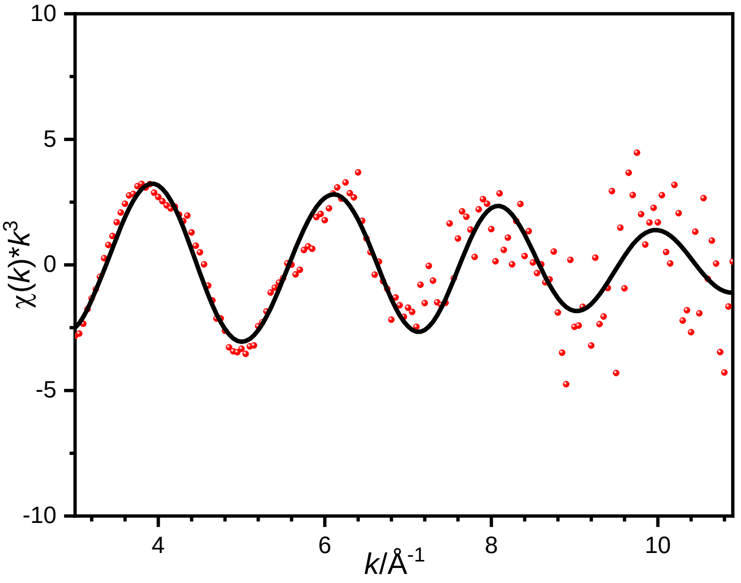

Supplementary Figure 21. Zn K-edge EXAFS (points) and the fitting curve (line) for ZnNC-900 catalyst, shown in *k*^{3}-weighted *k*-space.

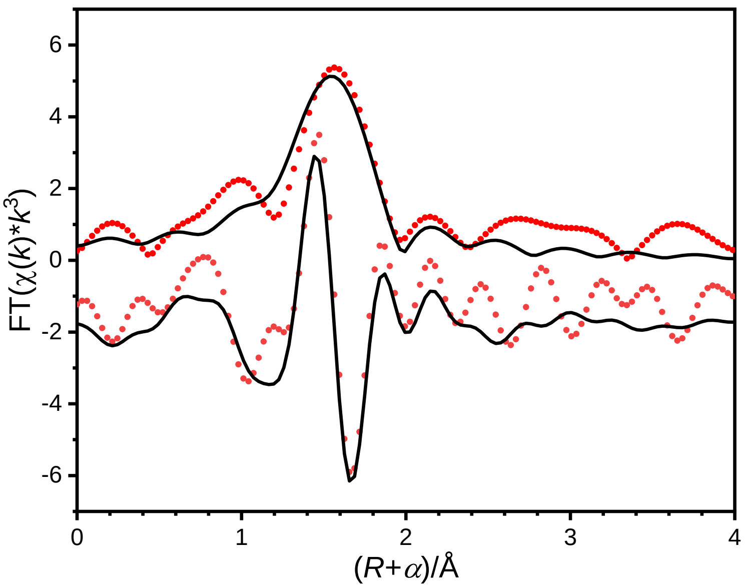

Supplementary Figure 22. Zn K-edge EXAFS (points) and the fitting curves (line) for ZnNC-900 catalyst, shown in *R*-space (FT magnitude and imaginary component). The dates are *k*^{3}-weighted and not phase-corrected.

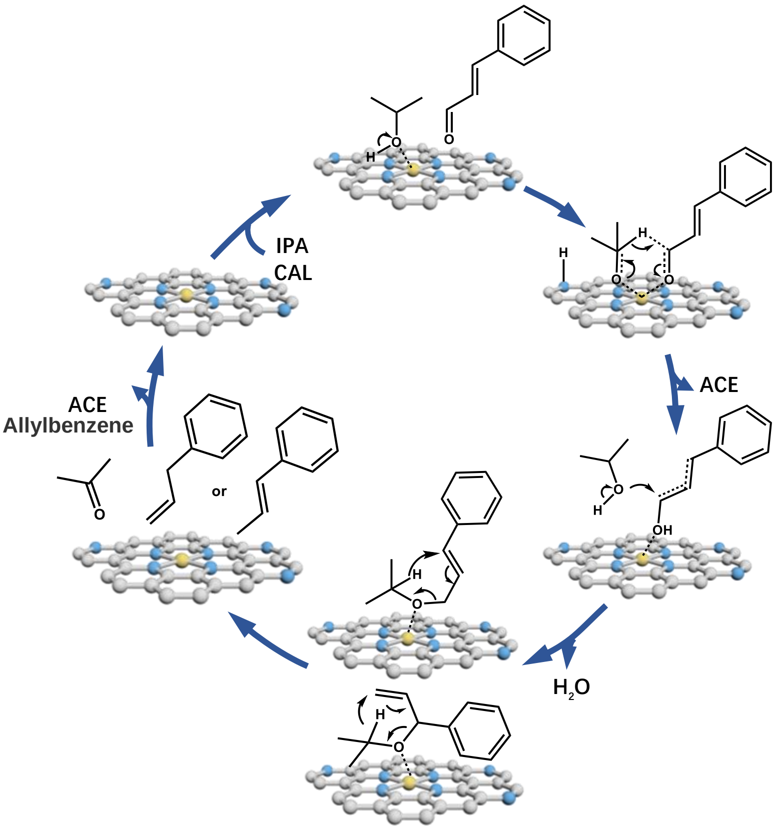

Supplementary Figure 23. The possible reaction mechanism. (Zn is yellow. N is blue. C is gray.)

Supplementary data (Gas chromatograms and mass spectra of the products by GC and GC-MS (EI))

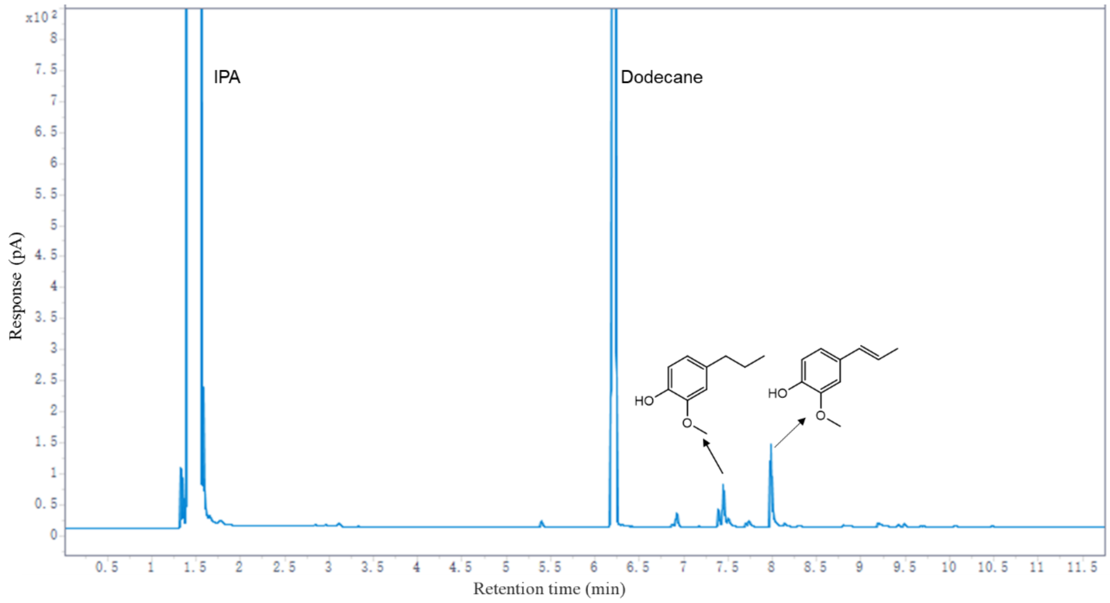

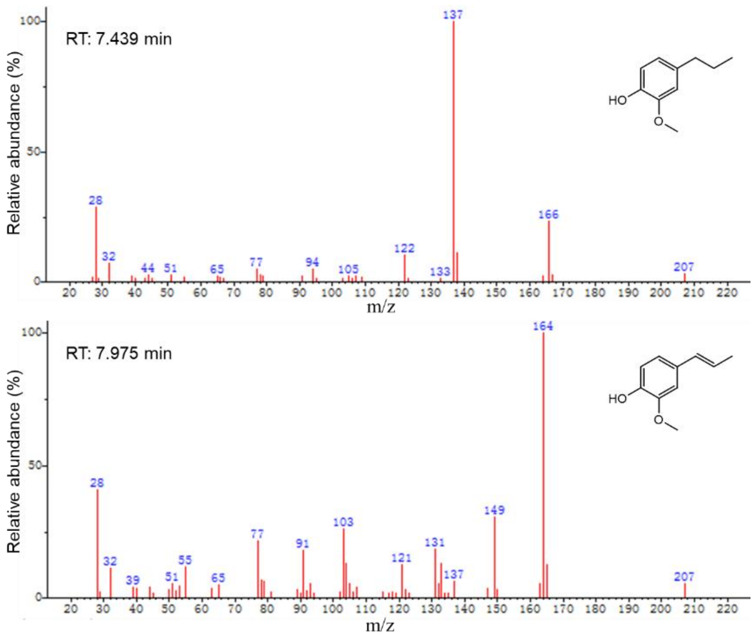
RT: 7.439 min
Relative abundance (%)
m/z
HO
O
RT: 7.975 min

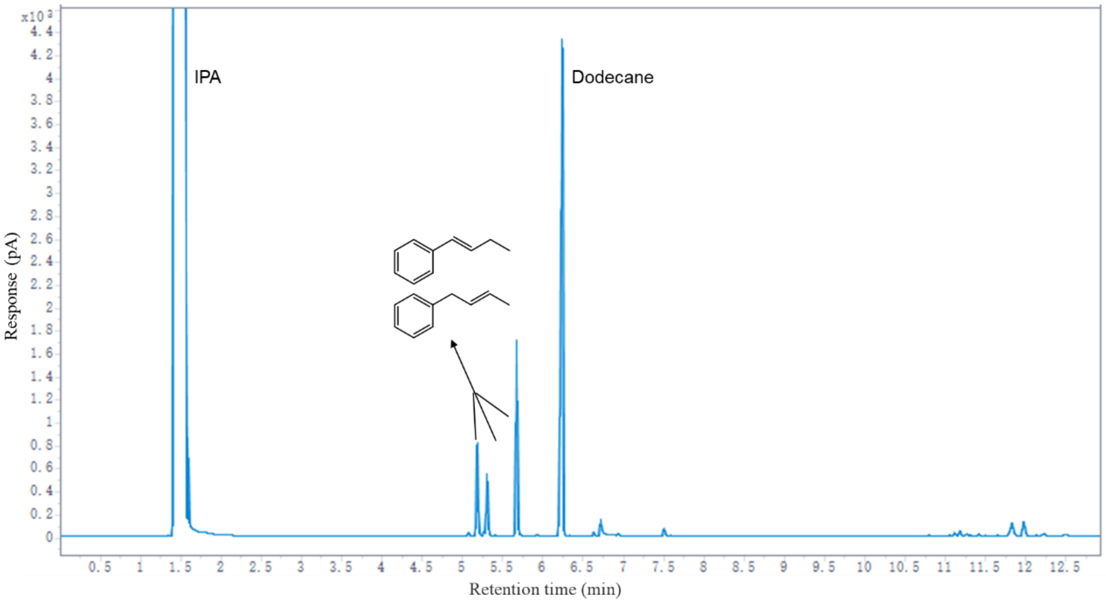
Response (pA)
Retention time (min)
IPA
Dodecane

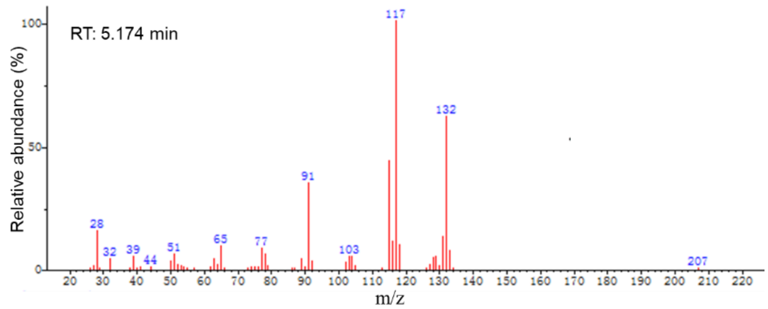
RT: 5.174 min
Relative abundance (%)
m/z
28
32
39
44
51
65
77
91
103
117
132
207

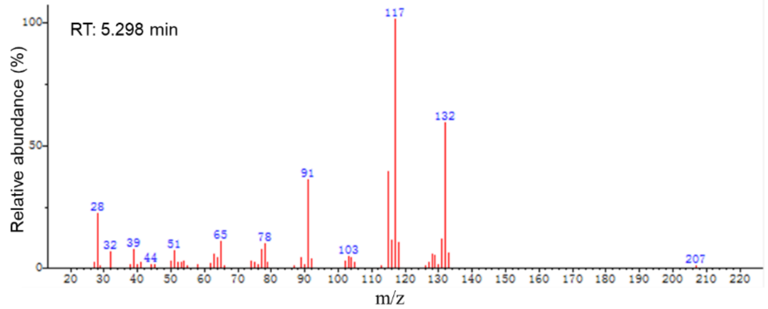
RT: 5.298 min
Relative abundance (%)
m/z
28
32
39
44
51
65
78
91
103
117
132
207

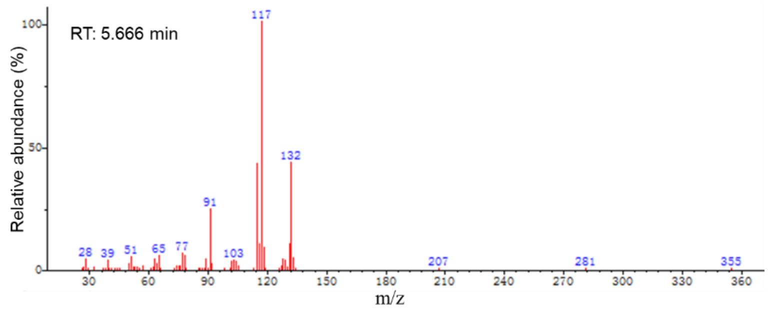
RT: 5.666 min
Relative abundance (%)
m/z
28
39
51
65
77
91
103
117
132
207
281
355

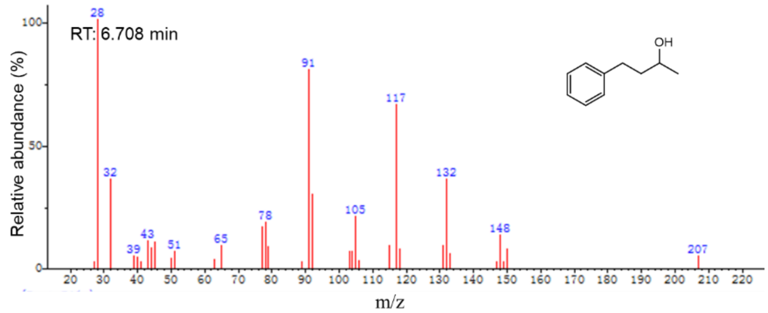
RT: 6.708 min
Relative abundance (%)
m/z
OH
28
32
39
43
51
65
78
91
105
117
132
148
207

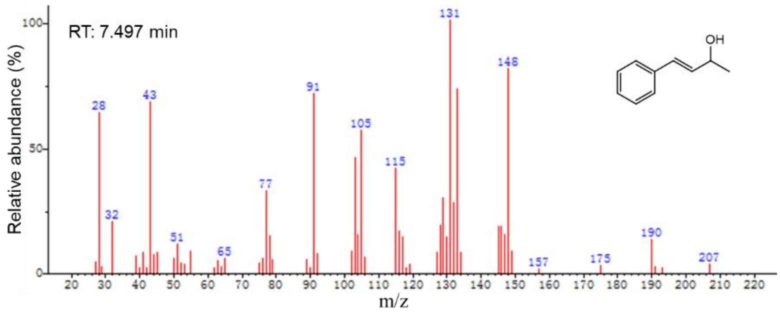
RT: 7.497 min
Relative abundance (%)
m/z
OH
28
32
43
51
65
77
91
105
115
131
148
157
175
190
207

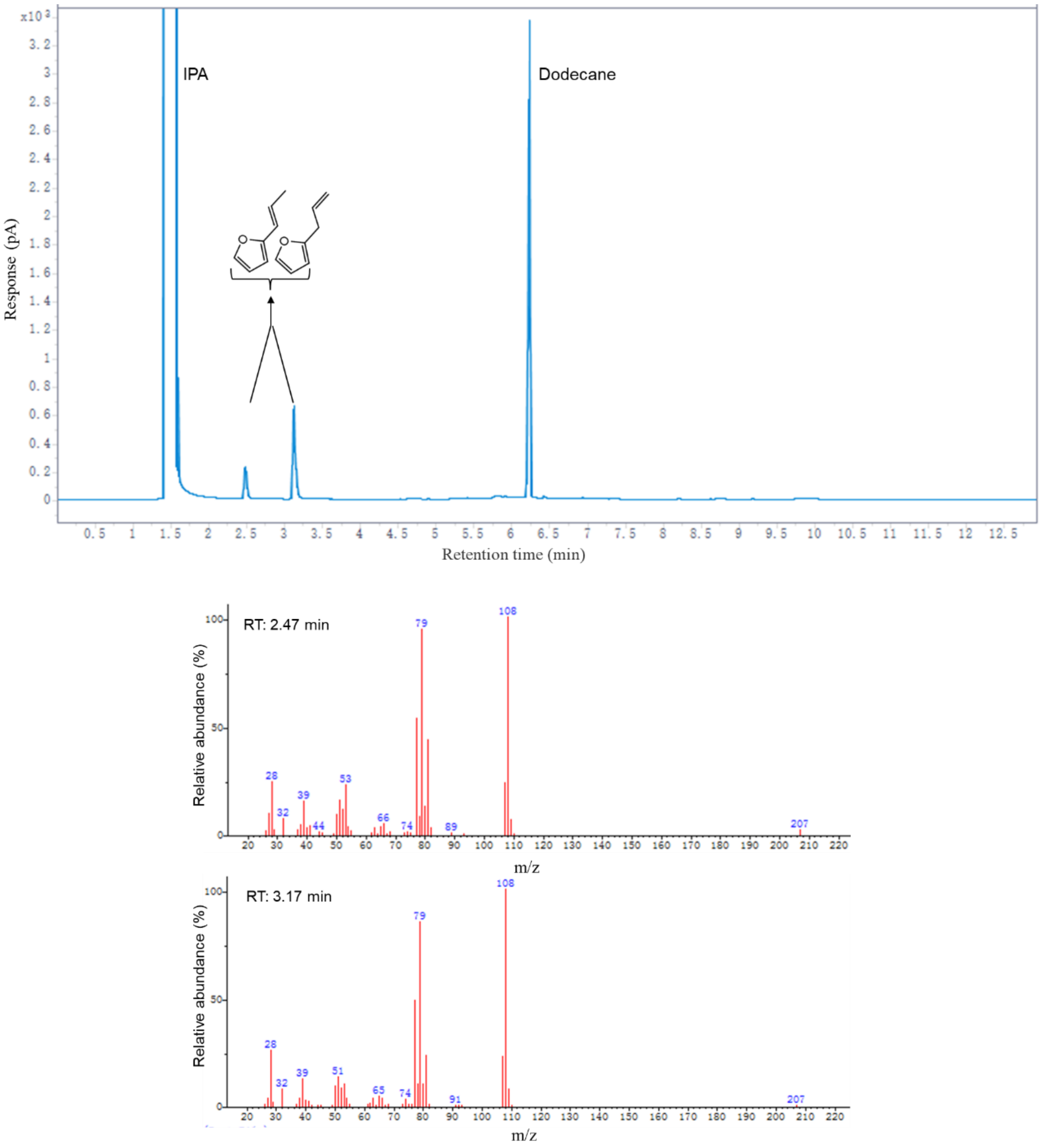

IPA
Dodecane
Response (pA)
Retention time (min)
RT: 2.47 min
Relative abundance (%)
m/z
RT: 3.17 min
Relative abundance (%)
m/z

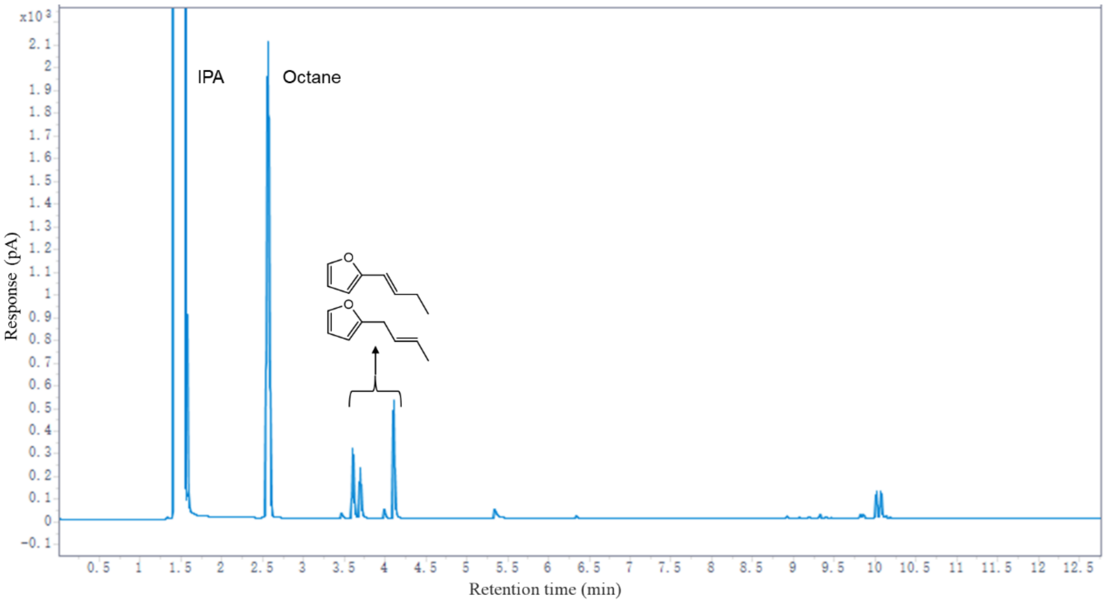
IPA
Octane
Response (pA)
Retention time (min)

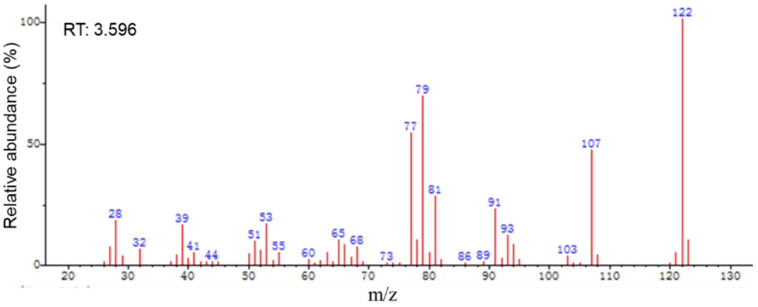
RT: 3.596
Relative abundance (%)
m/z

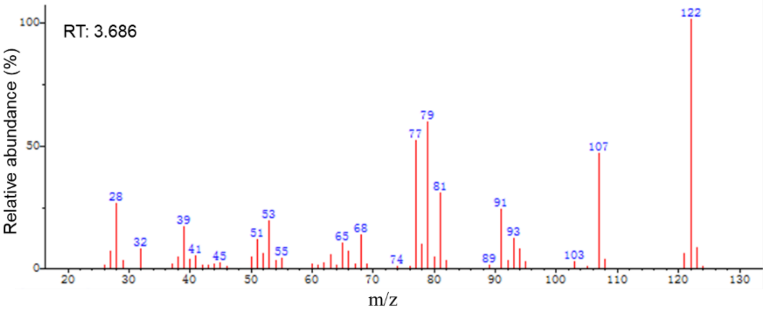
RT: 3.686
Relative abundance (%)
m/z

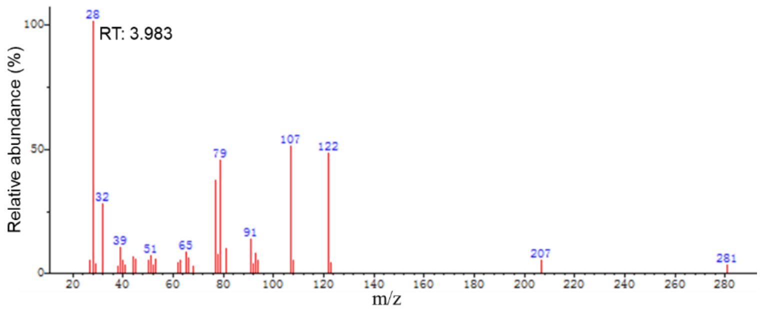
RT: 3.983
Relative abundance (%)
m/z

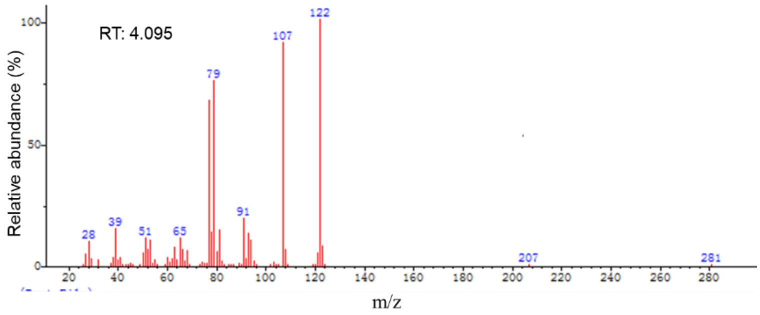
RT: 4.095
Relative abundance (%)
m/z

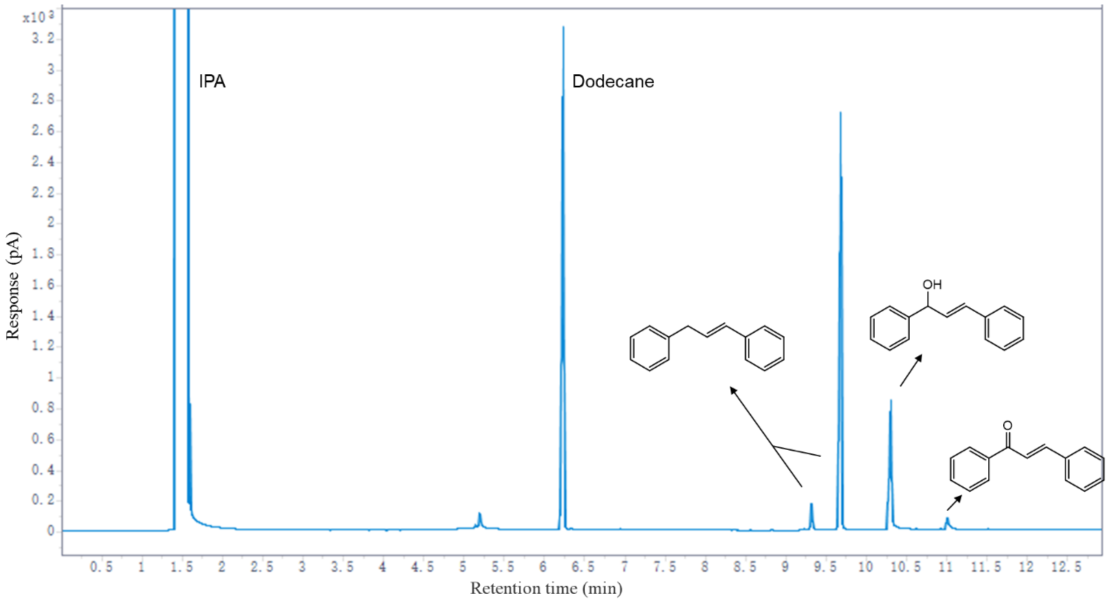
IPA
Dodecane
OH
O
Response (pA)
Retention time (min)

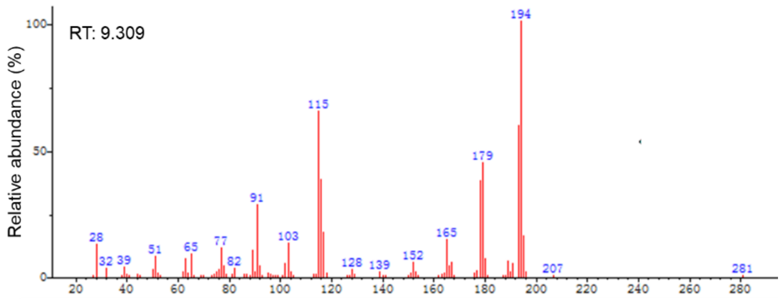
RT: 9.309
Relative abundance (%)
194
115
179
91
165
28
103
51
65
77
32
39
82
128
139
152
207
281

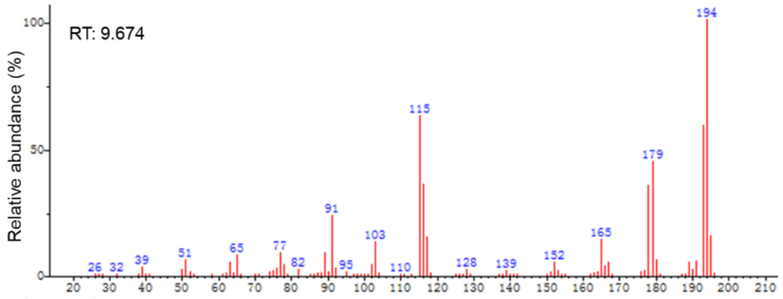
RT: 9.674
Relative abundance (%)
194
115
179
91
165
103
77
65
51
152
39
128
139
26
32
82
95
110

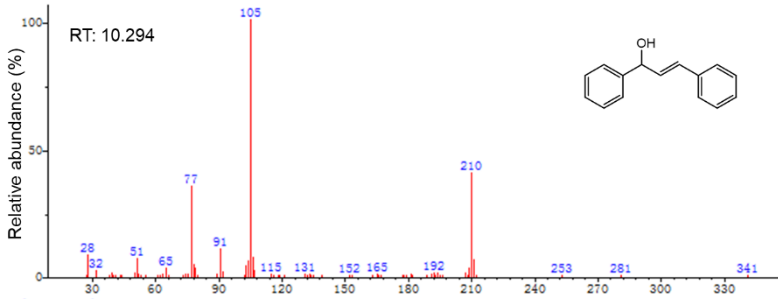
RT: 10.294
Relative abundance (%)
105
210
77
91
28
51
32
65
115
131
152
165
192
253
281
341
OH
